# Myelination of the brain in Major Depressive Disorder: An *in vivo* quantitative magnetic resonance imaging study

**DOI:** 10.1038/s41598-017-02062-y

**Published:** 2017-05-19

**Authors:** Matthew D. Sacchet, Ian H. Gotlib

**Affiliations:** 1 Department of Psychiatry and Behavioral Sciences, Stanford, CA 94305 USA; 2 Department of Psychology, Stanford University, Stanford, CA 94305 USA; 3 Neurosciences Program, Stanford University, Stanford, CA 94305 USA

## Abstract

Evidence from post-mortem, genetic, neuroimaging, and non-human animal research suggests that Major Depressive Disorder (MDD) is associated with abnormalities in brain myelin content. Brain regions implicated in this research, and in MDD more generally, include the nucleus accumbens (NAcc), lateral prefrontal cortex (LPFC), insula, subgenual anterior cingulate cortex (sgACC), and medial prefrontal cortex (mPFC). We examined whether MDD is characterized by reduced myelin at the whole-brain level and in NAcc, LPFC, insula, sgACC, and mPFC. Quantitative magnetic resonance imaging (qMRI) permits the assessment of myelin content, *in vivo*, in the human brain through the measure of R1. In this study we used qMRI to measure R1 in 40 MDD and 40 healthy control (CTL) participants. We found that the MDD participants had lower levels of myelin than did the CTL participants at the whole-brain level and in the NAcc, and that myelin in the LPFC was reduced in MDD participants who had experienced a greater number of depressive episodes. Although further research is needed to elucidate the role of myelin in affecting emotional, cognitive, behavioral, and clinical aspects of MDD, the current study provides important new evidence that a fundamental property of brain composition, myelin, is altered in this disorder.

## Introduction

Major Depressive Disorder (MDD) is a debilitating psychiatric disorder characterized by low levels of positive mood, high levels of negative mood, and loss of pleasure, or anhedonia^1^. Depression is a prevalent disorder, with an estimated 350 million individuals affected worldwide^2^, and is associated with a large and increasing economic, societal, and personal burden^3^. Despite the significant negative impact of MDD, its underlying pathophysiology is not well understood, and rates of treatment non-response and recurrence remain high^4, 5^. Neurobiological models of MDD have the promise to provide specific mechanistic insights regarding the etiology of depression that will inform targeted prevention and treatments, ultimately leading to a reduction in the prevalence of, and improved outcomes for, this debilitating disorder.

Depression is characterized by abnormalities in the brain, including myelin, a specialized, lipid-rich, electrically insulating tissue that is fundamental to neuron-to-neuron electrical communication. For example, postmortem studies have documented depression-related abnormalities, largely in the prefrontal cortex (PFC), in concentrations of proteins related to myelin, in transcription factors of myelin-related genes, and in glial cells that produce and maintain myelin refs 6 and 7. Studies of non-human animal models of MDD have similarly found reduced myelin, also in the PFC, in addition to reduced gene expression and altered morphology of myelin support cells, in this disorder^8, 9^.

Several quantitative magnetic resonance imaging (qMRI) methods have been used to delineate myelin-related properties of white matter in individuals with MDD. These methods include diffusion weighted imaging (DWI), which estimates myelin concentration from the amount of directional diffusivity (for review see refs 6, 10 and 11), and magnetization transfer imaging (MTI), which estimates myelin concentration based on the macromolecular content of the tissue^12–15^. The interpretation of findings obtained with these measures is limited, however, given that multiple biological properties can influence these non-specific signals, including intra-voxel orientation dispersion, axonal myelination, thickness, and density, and the presence of macrophages^16–23^. DWI and MTI are of particularly limited utility for quantifying myelin in gray matter, where myelin comprises only 5–10% of the macromolecular dry weight in gray matter, compared to 50% in white matter. Consequently, relations among myelin, directional diffusivity, and macromolecular content are likely to be even weaker in gray matter than in white matter^22, 24^.

R1 (1/T1) is a qMRI parameter that is a promising alternative to DWI and MTI for quantifying myelin *in vivo* using MRI. R1 quantifies the longitudinal relaxation rate of water hydrogen protons in a magnetic field and is sensitive to myelin^25, 26^. Researchers have documented high correlations between R1 and histological measures of myelin content in white matter (rs = 0.70–0.89)^27–29^, and there is no *a priori* reason to believe that there would not also be a strong relation between myelin and R1 in gray matter^30^. Indeed, Stüber *et al*. recently found that R1 signal contrast is fully dependent on a linear combination of myelin and iron concentrations in both white and gray matter, with the predominant signal being contributed from myelin (myelin/iron signal contribution: white matter = 90%/10%, gray matter 64%/36%)^31^. Given prior research suggesting that iron content is increased in the brains of individuals with MDD^32^, it is likely that *decreases* in R1 are attributable to decreases in myelin. In this context, investigators have begun to use R1 as an *in vivo* assay of myelin content in gray and white matter (for review see ref. 30).

The NAcc, lateral prefrontal cortex (LPFC), insula, subgenual anterior cingulate cortex (sgACC), and medial prefrontal cortex (mPFC) have been implicated in the pathophysiology of MDD, and have been associated with key clinical and psychological features of this disorder. Dysfunctional reward processing is a significant component of MDD^33^; indeed, anhedonia is one of the two core symptoms of MDD^1^. The NAcc has been implicated in anomalous reward processing in MDD and is posited to underlie motivation-related abnormalities in this disorder^34–40^. Postmortem studies of depressed humans^6, 7^ and studies of animal models of MDD^8, 9^ have documented reduced myelin in the LPFC this disorder. The LPFC is a core node of the cognitive control network and has been implicated in a broad array of cognitive control processes, including working memory, behavioral inhibition, and attention allocation. In this context, a large body of research has found that depressed individuals are characterized by reduced cognitive control (for meta-analytic review see ref. 41), and neuroimaging studies have found abnormal activity of the LPFC in MDD (for a meta-analytic review see ref. 42). Furthermore, MDD is characterized by rumination^43, 44^ (i.e., negative automatic thoughts focused on the self that may be driven by abnormal cognitive control) and, therefore, may involve the LPFC. The insula is a core node of the ventral attention network (also referred to as the salience network)^45^ and is posited to be involved in the awareness of, and the orientation and response to, relevant stimuli and interceptive states^46, 47^. In fact, the insula has been implicated in heightened processing of negative material in MDD (for meta-analytic results see ref. 42). The default mode network (DMN) is implicated in self-focused mentation and is thought to be involved in ruminative processes in MDD. The mPFC is a core node of the DMN; activity in this region has been found to be related to levels of ruminative self-focus in MDD^48^. Another study reported that functional connectivity between mPFC and sgACC was significantly related to depressive rumination^49^. Although not part of the DMN in healthy individuals, the results of several studies suggest that the sgACC is coactive with thƒe DMN in individuals with MDD^50, 51^, and other researchers have found relations between sgACC activity and rumination^52, 53^. More generally, the sgACC may underlie feelings of sadness in MDD^47, 54, 55^; moreover, sgACC activity has been shown to predict treatment response in this disorder^56^.

In this study we compared *in vivo* myelin concentration in individuals diagnosed with MDD and in healthy control participants (CTLs), using qMRI to compute R1. We tested the following hypotheses: 1) myelin concentration is abnormal in MDD at the whole-brain level; 2) myelin content of the NAcc, LPFC, insula, sgACC, and mPFC are abnormal in MDD; 3) myelin content of the NAcc is associated with anhedonia and myelin content of the LPFC, sgACC, and mPFC are associated with rumination; and 4) myelin content in these three regions, LPFC, insula, and at the whole-brain level, is associated with depression severity and the number of prior episodes of MDD.

## Results

### Participant characteristics

Demographic and clinical characteristics of the depressed and nondepressed participants are presented in Table 1. The two groups did not differ with respect to age, gender, handedness, income, level of education, race, lateral ventricle volume, brain segmentations without ventricles, or eICV (all *ps* > 0.18). As expected, compared with the CTL participants, the MDD participants had higher levels of depression, anhedonia, and rumination (*p*

**Table 1:** Participant characteristics.

	MDD				CTL				Statistic		p-value	Effect size
N	39				40							
Age (M|SE|t)	37.0		2.1		35.1		1.9		0.67		0.506	0.15
Gender (Male; N|%|χ^2^)	14		35.9		13		32.5		0.10		0.750	0.04
Handedness (R|L|A|χ^2^)	35	3		1	30	9		1	3.37		0.185	0.21
Psychotropic Medication use (N|%)	11		28.2%		0		0.0					
Anxiety Disorder Comorbidities (N|%)	22		56.4%		0		0.0					
Number of depressive episodes (Med|IQR)	5		8.75		0		0.0					
BDI-II Scores (M|SE|t)	29.5		2.0		2.5		0.5		13.43		<0.001	3.00
BDI-II Anhedonia (Med|IQR|rank sum|z-score)	3		2		0		0		2244.5	7.07	<0.001	0.80
RRS: Reflection Subscale (M|SE|t)	13.2		0.5		7.5		0.5		8.50		<0.001	1.91
RRS: Brooding Subscale (M|SE|t)	13.8		0.6		6.5		0.2		12.16		<0.001	2.73
Annual Income (Med|IQR|rank sum|z-score)	3		4		3		2		1395.5	0.67	0.504	0.08
Education (Med|IQR|rank sum|z-score)	7		1.75		7		1.5		1692.0	0.95	0.342	0.11
Race (χ^2^)	N		%		*N*		%		4.37		0.358	0.24
Asian	5		12.8%		11		12.5%					
Black/African American	3		7.7%		2		5.0%					
Native Hawaiian/Pacific Islander	1		2.6%		0		0.0%					
White/Caucasian	26		66.7%		21		52.5%					
Other/multiracial	4		10.3%		6		15.0%					
Lateral ventricle volume (mm^3^; M|SE|t)	6407.7		494.5		5955.3		584.3		0.59		0.557	0.13
Brain segmentation (mm^3^; M|SE|t)	1.06*10^6^		1.38*10^4^		1.05*10^6^		1.58*10^4^		0.21		0.836	0.05
eICV (mm^3^; M|SE|t)	1.40*10^6^		2.04*10^4^		1.38*10^6^		2.27*10^4^		0.84		0.405	0.19

Number of depressive episodes was set to 10 when participant indicated non-specific large values (e.g., “too many to count”). Income was coded from 0 to 5 (<$10,000; $10,000–25,000; $25,000–50,000; $50,000–75,000; $75,000–100,000; >$100,000). Education was coded from 0–4 (some college; technical school; junior college; four-year college; graduate or professional degree). Income was unknown for 7 individuals in both groups. MDD = Major Depressive Disorder group; CTL = healthy control group; *N* = number participants; *M* = mean; *SE* = standard error; *t* = *t*-statistic (effec*t* sizes computed as Cohen’s *d*); *χ* ^2^ = chi-square statistic (effect sizes computed as *φ*); rank sum = Wilcoxon rank sum statistic; *z*-stat = normal statistic (effect sizes were computed from rank sum *z*-scores as *z*-score/√(*N*_MDD_ + *N*_CTL_)); R = right; L = left; A = ambidextrous; BDI-II = Beck Depression Inventory-II^72^; BDI-II Anhedonia = anhedonia factor of the BDI-II^72–74^; RRS = Ruminative Response Styles (RRS) scale^87^; Med = median; IQR =interquartile range.

< 0.001).

### Assessment of differences in R1 between depressed and nondepressed participants

The depressed participants had significantly lower whole-brain R1 levels than did the nondepressed participants (*t*(77) = 2.157, *p* = 0.034, Cohen’s *d* = 0.48; *M*/*SE*: MDD 0.760/0.003, CTL 0.768/0.003). GLMs conducted using group as a between-subjects factor and hemisphere as a within-subject factor indicated that the MDD group had significantly lower levels of R1 in the NAcc, LPFC, sgACC, and medial superior extrastriate visual network control region than did the CTL group; however, only the effect for the NAcc remained significant after covarying for whole-brain R1 (*F* = 5.89, *p* = 0.018, partial *η* ^2^ = 0.07; see Table 2 for all results). None of the GLMs yielded significant main effects of hemisphere or significant interactions of whole-brain R1 and hemisphere or group after covarying for whole-brain R1 (*F*s ≤ 3.49, ps ≥ 0.066, partial *η* ^2^s ≤ 0.04). For the LPFC, insula, sgACC, mPFC, and visual control network ROIs there was a significant effect of the whole-brain R1 covariate, indicating that whole-brain R1 was significantly related to R1 in each of these ROIs (*F*s ≥ 18.50, *ps* ≤ 0.001, partial *η* ^2^s ≥ 0.20); whole-brain R1 was not significantly related to NAcc R1 (*F* = 0.68, *p* = 0.412, partial *η* ^2^ = 0.01).

**Table 2:** Regional assessment of R1. General linear models repeated over hemisphere were used to assess the effect of group in regional R1.

ROI	MDD		CTL		*F*	*p*-value	Partial *η* ^2^
	*M/EMM*	*SE*	*M/EMM*	*SE*			
Without whole-brain R1 covariate							
NAcc	0.635	0.004	0.650	0.004	7.33	0.008	0.09
LPFC	0.734	0.004	0.745	0.004	4.40	0.039	0.05
Insula	0.628	0.002	0.634	0.002	3.62	0.061	0.05
sgACC	0.610	0.004	0.625	0.004	5.69	0.019	0.07
mPFC	0.726	0.004	0.734	0.004	1.57	0.214	0.02
Lateral striate^a^	0.665	0.009	0.675	0.009	0.65	0.421	0.01
Lateral extrastriate^a^	0.718	0.005	0.726	0.005	1.38	0.244	0.02
Medial striate^a^	0.740	0.005	0.749	0.005	2.02	0.160	0.03
Medial inferior extrastriate^a^	0.641	0.005	0.647	0.005	0.80	0.374	0.01
Medial superior extrastriate^a^	0.681	0.005	0.695	0.005	4.22	0.043	0.05
Whole-brain R1 covariate							
NAcc	0.636	0.004	0.649	0.004	5.89	0.018^b^	0.07
LPFC	0.738	0.003	0.741	0.003	0.59	0.445	0.01
Insula	0.630	0.002	0.632	0.002	0.86	0.358	0.01
sgACC	0.614	0.004	0.622	0.004	2.15	0.147	0.03
mPFC	0.731	0.003	0.729	0.003	0.35	0.555	0.01
Lateral striate^a^	0.672	0.008	0.668	0.008	0.10	0.758	0.00
Lateral extrastriate^a^	0.723	0.004	0.721	0.004	0.04	0.848	0.00
Medial striate^a^	0.745	0.003	0.744	0.003	0.01	0.920	0.00
Medial inferior extrastriate^a^	0.644	0.004	0.644	0.004	0.00	0.949	0.00
Medial superior extrastriate^a^	0.686	0.004	0.690	0.004	0.65	0.421	0.01

MDD = Major Depressive Disorder group; CTL = healthy control group; R = right; L = left; ROI = region of interest; *M*/*EMM* = mean or estimated marginal mean, EEM computed when covarying for whole-brain R1; *SE* = standard error; *F* = general linear model *F*-statistic for the effect of group; LPFC = lateral prefrontal cortex; sgACC = subgenual anterior cingulate cortex; mPFC = medial prefrontal cortex; ^a^ = visual network control regions; ^b^ = when including the whole-brain R1 covariate only the NAcc exhibited a significant effect of group.

### Number of prior episodes of depression and R1

Nine of the 39 MDD participants indicated that they had experienced too many previous depressive episodes to count accurately; therefore, we assigned a value of 10 on this measure to these participants and conducted a median split of the MDD group on the number of previous episodes experienced. This procedure yielded a subgroup of MDD participants who had experienced 5 or fewer depressive episodes (*N* = 22) and a subgroup who had experienced 6 or more episodes (*N* = 17). These MDD subgroups did not differ with respect to age (*t*(37) = 1.25, *p* = 0.219, Cohen’s *d* = 0.40). We conducted GLMs to compare R1 in these two subgroups, and further examined significant between-group differences by comparing each subgroup with the CTL group.

The two MDD subgroups did not differ in R1 at the whole-brain level, in the NAcc, insula, sgACC, or in the visual network control regions. The MDD subgroup with more depressive episodes exhibited lower R1 in both the LPFC and mPFC than did the MDD subgroup with fewer depressive episodes (LPFC: *t*(37) = 2.11, *p* = 0.042, Cohen’s *d* = 0.68; mPFC: *t*(37) = 2.08, *p* = 0.045, Cohen’s *d* = 0.67). Subsequent GLMs conducted with whole-brain covariates indicated that the more depressive episodes MDD subgroup also had lower LPFC R1 than did the CTL group (*F*(1) = 4.24, *p* = 0.044, partial *η* ^2^ = 0.07), while CTL group R1 did not differ significantly from the fewer depressive episodes MDD subgroup (*F*(1) = 0.23, *p* = 0.633, partial *η* ^2^ = 0.00). Neither the MDD subgroup with more depressive episodes nor the MDD subgroup with fewer depressive episodes differed significantly from the CTL group in R1 in mPFC group (more episodes subgroup: *F*(1) = 1.20, *p* = 0.279, partial *η* ^2^ = 0.02; fewer episodes subgroup: *F*(1) = 3.37, *p* = 0.072, partial *η* ^2^ = 0.05). See Table 3 for all results comparing more and less episodes MDD subgroups.

**Table 3:** R1 and the number of depressive episodes of Major Depressive Disorder. Median split was used to create two groups of MDD participants based on the number of prior depressive episodes. The more episodes group included individuals with 6 or more episodes (*N* = 17), and the fewer group 5 or less episodes (*N* = 22).

ROI	More Episodes		Fewer Episodes		*t-*statistic	*p*-value	Cohen’s *d*
	*M*	*SE*	*M*	*SE*			
Whole-brain	0.761	0.005	0.758	0.004	0.57	0.573	0.19
NAcc	0.635	0.005	0.636	0.006	0.12	0.904	0.04
LPFC	0.725	0.006	0.741	0.005	2.12	0.042^b^	0.68
Insula	0.623	0.003	0.632	0.003	1.82	0.077	0.58
sgACC	0.613	0.008	0.608	0.007	0.49	0.624	0.16
mPFC	0.716	0.006	0.734	0.006	2.08	0.045^b^	0.67
Lateral striate^a^	0.671	0.016	0.660	0.014	0.52	0.606	0.16
Lateral extrastriate^a^	0.714	0.010	0.720	0.008	0.51	0.610	0.16
Medial striate^a^	0.736	0.006	0.743	0.006	0.83	0.410	0.27
Medial inferior extrastriate^a^	0.636	0.007	0.644	0.006	0.90	0.375	0.30
Medial superior extrastriate^a^	0.676	0.008	0.684	0.007	0.80	0.430	0.26

ROI = region of interest; *M* = mean; *SE* = standard error; NAcc = nucleus accumbens; LPFC = lateral prefrontal cortex; sgACC = subgenual anterior cingulate cortex; mPFC = medial prefrontal cortex; ^a^ = visual network control regions; ^b^ = significant at *p* < 0.05.

### R1 and clinical and psychological characteristics of the depressed participants

We assessed relations between R1 and clinical and psychological characteristics of the depressed participants. Whole-brain R1 levels were not related significantly to severity of depression (*r* = −0.10, *p* = 0.551). NAcc R1 was not correlated with either anhedonia (Spearman *r* = −0.22, *p* = 0.179) or severity of depression (*r* = −0.282, *p* = 0.082). LPFC R1 in the more depressive episodes MDD subgroup was not significantly related to depression severity or to rumination (Beck Depression Inventory-II [BDI-II]: *r* = −0.07, *p* = 0.782; Ruminative Response Styles [RRS] reflection: *r* = 0.40, *p* = 0.109; RRS brooding: *r* = −0.11, *p* = 0.671). Moreover, across all MDD participants LPFC R1 was not significantly related to depression severity or to rumination (BDI-II: *r* = −0.02, *p* = 0.918; RRS reflection: *r* = 0.12, *p* = 0.470; RRS brooding: *r* = 0.00, *p* = 0.997). Insula, sgACC, and mpFC R1 were not correlated with severity of depression (all |*r*| ≤ 0.10, all *p* ≥ 0.549). Neither sgACC nor mPFC R1 was correlated with scores on the brooding subscale of the RRS (sgACC: *r* = 0.26, *p* = 0.112; mPFC: *r* = 0.12, *p* = 0.482); mPFC R1 was also not correlated with scores on the reflection subscale of the RRS (*r* = 0.21, *p* = 0.199). R1 of sgACC was positively correlated with scores on the reflection subscale of the RRS (*r* = 0.45, *p* = 0.004). Finally, no relations were found between mPFC R1 and scores on the BDI-II or on the brooding or reflection subscales of the RRS within either MDD subgroup with more or fewer prior depressive episodes (all |*r*| ≤ 0.31, all *p* ≥ 0.162).

### Exploratory analyses relating psychotropic medication use and anxiety comorbidity to R1

We conducted exploratory analyses to assess the effects of psychotropic medication use and anxiety comorbidity on whole-brain, NAcc, and LPFC R1. The 11 medicated MDD participants had lower whole-brain R1 than did the 28 unmedicated MDD participants; no other medication-related differences were significant. The medicated and unmedicated subgroups did not differ with respect to either the severity of depression or the number of prior depressive episodes. The subgroup of MDD participants with comorbid anxiety (*N* = 22) did not differ from the subgroup of MDD participants without comorbid anxiety (*N* = 17) with respect to levels of whole-brain, NAcc, LPFC, insula, or mPFC R1. The MDD subgroup with comorbid anxiety disorder had higher sgACC R1 than did the MDD subgroup without comorbid anxiety disorder. The subgroup of MDD participants with comorbid anxiety was more severely depressed than was the MDD subgroup without comborbid anxiety, but the groups did not differ significantly with respect to the number of prior episodes. See “Supplementary Results” and Tables S1 and 2 for further detail.

### Exploratory analyses assessing LPFC subregions

We conducted exploratory analyses to assess group differences in R1 in subregions of LPFC. There was a significant effect of group only in the left dorsal LPFC subregion, which was no longer significant after covarying for whole-brain R1 (Supplementary Information Table S3).

We conducted additional exploratory analyses to assess differences between the two subgroups of MDD participants in R1 in subregions of LPFC. Only the dorsal subregion of left LPFC yielded a group difference: the ‘more episodes’ MDD subgroup had lower R1 than did the ‘fewer episodes’ subgroup (*t*(37) = 2.31, *p* = 0.027, Cohen’s *d* = 0.77; See Supplementary Information Table S4 for full results). Subsequent GLMs covarying for whole-brain R1 indicated that the ‘more episodes’ MDD subgroup also had lower R1 than did the CTL group (*F*(1) = 7.84, *p* = 0.007, partial *η* ^2^ = 0.13; *EMM*/*SE*: ‘more episodes’ MDD = 0.656/0.009; CTL = 0.688/0.006); the ‘fewer episodes’ MDD subgroup did not differ from the CTL group in R1 of the left dorsal subregion of LPFC (*F*(1) = 0.04, *p* = 0.850, partial *η* ^2^ = 0.00; *EMM*/*SE*: ‘fewer episodes’ MDD = 0.685/0.009; CTL = 0.687/0.006).

### Relations between R1 and age

We used permutation tests to assess whether the MDD and CTL groups exhibited significantly different relations between age and R1 in NAcc, LPFC, insula, sgACC, and mPFC. There were no group differences in relations between age and R1 in any of these regions (permutation test: all *p*s ≥ 0.254).

## Discussion

This study was designed to use R1 to assess levels of myelin in the brains of individuals with MDD. We found that MDD participants were characterized by reduced R1 at the whole-brain level compared to CTLs. Using ROI analyses, we found that NAcc R1 was reduced in MDD, and that the number of prior episodes of MDD was associated with reductions in R1 in LPFC, which were particularly prominent in a left dorsal subregion of LPFC. These findings offer new and important evidence that MDD is characterized by abnormalities in myelin content.

The biological mechanisms that underlie abnormal myelin content in MDD are not well understood^57^. One proposed mechanism is based on the formulation that myelination is an adaptive process that is dependent on environmental influence through axonal firing rate^58^. A growing literature indicates that neuronal activity increases myelination; indeed, optimal neuronal function depends on a bidirectional influence between axons and myelin^57, 59, 60^. In the context of MDD and the PFC, social isolation has been shown to reduce myelin content in the PFC in mice^8, 9, 61^. Liu *et al*. interpreted this finding as the socially isolated mice not activating their PFC. More broadly, it is widely documented that rodents exposed to stress exhibit reduced neuronal activity in PFC, and that this results in depression-related behaviors^61–63^. Covington *et al*. used optogenetics to stimulate the PFC, that resulted in a reduction of depression-related behaviors, including behaviors related to social interaction and anhedonia^63^. Relatedly, optogenetic stimulation of mouse premotor cortex has been shown to result in myelination^64^, and drug-induced myelination of PFC using clemastine has resulted in reduced depression-related behavior in mice^8^. Taken together, myelination and axonal firing influence each other; thus, reduced activity of axonal firing may result in reductions in myelin.

A second, related, mechanism that may explain relations between reduced myelination and MDD involves biological pathways that are affected by stress, resulting in neuroinflammation and adverse effects to myelin. More generally, inflammation has been posited to contribute to MDD^65, 66^. Relevant evidence includes findings that individuals with MDD have higher levels of pro-inflammatory cytokines, including interleukin 1β (IL-1β), interleukin 6 (IL-6), and tumor necrosis factor alpha, than do their nondepressed counterparts^67, 68^. In this context, stress promotes an inflammatory response through multiple different mechanisms that result in the release of proinflammatory cytokines. Cytokines are small signaling proteins released by cells that affect the function of other cells. Cytokines act on a variety of biological substrates, and after prolonged exposure, can result in damage to myelin. In this context, in MDD, stress, and associated neuroinflammation, particularly over prolonged periods, may result in widespread damage to myelin in the brain. Indeed, pro-inflammatory cytokines have been shown to cause demyelination in cerebellar cultures^69^, and myelin sheath-related abnormalities, including loss of vacuolization, vesiculation, demyelination, swelling of water gaps, loss of adhesion, and lesion formation, are fundamental characteristics of inflammatory diseases of the brain^70^. In sum, abnormal reductions in myelin content in MDD may be due to decreased axonal activity in specific brain regions^8, 58^, and/or to stress-induced neuroinflammation and cytokines^65^. Although we did not directly assess the mechanisms driving reduced myelin content in MDD, below we discuss the current findings in the context of these proposed mechanisms.

The finding that whole-brain measures of R1 were reduced in MDD suggests that there are global reductions in myelination in the brains of depressed individuals. Importantly, we obtained this result in a comparison of MDD and CTL groups that did not differ in whole-brain or ventricular volume, indicating that reduced whole-brain myelin in MDD is not related to anomalous ventricular or whole-brain volumes. The finding that R1 is reduced globally in MDD, however, tells us little regarding the regional specificity, if any, of this abnormality. That is, it is possible that this effect is driven by small, widespread reductions in R1 or by large reductions in a small number of brain regions. In this context, when we controlled for whole-brain R1 in regional analyses, the group-related effects in the LPFC, sgACC, and the medial superior extrastriate visual network control region were no longer statistically significant, suggesting that reductions in R1 in MDD in relatively distant brain regions can be explained by global reductions in R1. That said, however, there was not a significant effect of group in the insula, mPFC, or four other visual network control regions, even when we did not control for whole-brain R1. Moreover, R1 in the NAcc was reduced in the MDD compared to the CTL group, over and above the effects of abnormalities in whole-brain R1. Thus, it appears that the brains of depressed individuals exhibit both widespread and regionally specific reductions in R1.

The widespread abnormalities in myelin found in the current study suggest that the whole brain exhibits reduced activity in MDD, and/or that neuroinflammation-related cytokines negatively affect large proportions of the brain. Evidence for widespread neural abnormality in MDD includes a recent study from our group in which we found large-scale hypoconnectivity in a range of resting-state networks, including dorsal attention, default mode, and frontoparietal networks, in adolescent depression^71^. With respect to neuroinflammation, whole-brain reductions in myelin could be explained by a generalized pro-inflammatory cytokine-related insult to the brain. Future research should assess directly relations between global R1 and measures of large-scale brain activity and inflammatory cytokines.

NAcc R1 was reduced in the MDD compared to CTL group over and above the effects of global R1 differences. Given the role of the NAcc in reward processing and motivation, the observed myelination reduction in the NAcc in MDD may underlie anhedonia and pathological motivation, fatigue, and levels of energy. To assess this possibility, we tested whether anhedonia was correlated with NAcc R1 in the MDD group. This analysis did not yield a statistically significant correlation. One reason for this may be that the measure of anhedonia we used in this study, the BDI-II anhedonia factor^72–74^, is composed of only two items (“Loss of Pleasure” and “Loss of Interest”). Assessing anhedonia using a more comprehensive measure of this construct may yield greater variability and a stronger examination of the relation between NAcc R1 and anhedonia.

Studies examining anomalies in the NAcc in MDD have been inconsistent^75^. Findings of studies in this area include MDD-related reductions in NAcc activation during the anticipation of reward^76^, during the receipt of reward^77–79^, during both anticipation and receipt of reward, and during neither anticipation nor receipt of reward^35^. This lack of reliability in reward-related functional activations in MDD may be due to imaging-related nuisance variables (e.g., movement), or to neurobiological heterogeneity of MDD^75^. Misaki *et al*. have shown that variability in reward-related processing can be subtyped using unsupervised machine learning. In this context, it would be informative to examine the association of myelination of the NAcc with this these subtypes. We speculate that the subtype with the lowest activation in the NAcc during reward processing would exhibit the greater reductions in NAcc myelination, a pattern that would be consistent with the formulation that myelin content in a given region mirrors the amount of activity associated with that region^58, 80^. Alternatively, given that neuroinflammation damages myelin^65^ and has been shown to particularly affect the NAcc^81, 82^, it is possible that neuroinflammation underlies NAcc myelin abnormalities in this disorder. Future research should test this formulation directly.

LPFC R1 was found to be reduced in those depressed individuals who had experienced a greater number of previous depressive episodes. Reduced R1 in these individuals was confined to the LPFC; we did not find reduced R1 in the NAcc, insula, sgACC or at the whole-brain level. We did find that depressed individuals with more prior depressive episodes were characterized by lower levels of R1 in mPFC than were depressed individuals with fewer prior depressive episodes; neither of these subgroups, however, differed from the CTL group in mPFC R1. Moreover, the full MDD group did not have reduced R1 in the LPFC compared to the CTL group, suggesting that reduced LPFC myelination is associated specifically with the number of prior episodes of depression. Based on the formulation that myelination parallels activity in a given region^58^, this depressive episode-related reduction in LPFC R1 suggests that the LPFC becomes less active with more episodes of MDD. The specificity of this finding may also be explained by the LPFC being particularly susceptible to usage-dependent myelination because it develops relatively late compared to the rest of the brain^80, 83^. This possibility is also consistent with the reliable finding of abnormally reduced activity in LPFC in MDD (for meta-analytic review see ref. 42).

Because the LPFC is implicated in cognitive control, it is noteworthy that several studies have assessed, in older adults, earlier-onset MDD compared to later-onset MDD. The results of these studies have been mixed (for review see ref. 41). These inconsistencies may be driven by variability in LPFC R1, with greater reductions in LPFC R1 related to poorer performance on tasks assessing executive functioning. Furthermore, exploratory analyses in this study revealed that R1 in the left dorsal LPFC subregion is most strongly related to the number of prior episdoes of MDD. Future research should examine functional correlates of the abnormally reduced R1 in LPFC and the left dorsal subregion of LPFC. These studies might examine directly the relation between levels of R1 and functions associated with LPFC, focusing on cognitive control-related processes such as attention allocation and decision making.

We found that within the MDD group sgACC R1 was positively correlated with scores on the RRS Reflection subscale. Prior research has found that sgACC activity is positively correlated with levels of self-reported depressive rumination^49, 52, 53^. Our finding is, in part, consistent with the formulation that increased rumination leads to greater activity of sgACC and, consequently, to increases in myelination as a result of usage-dependent plasticity^58, 84, 85^. Not consistent with this interpretation, however, is the fact that we did not find increased R1 in the MDD compared to the CTL group, when the MDD group had significantly higher scores on the RRS Reflection subscale than did the CTL group (*t* = 8.50, *p* < 0.001, Cohen’s *d* = 1.91). Future research is needed to gain a better understanding of the nature of the relation between sgACC R1 and ruminative reflection.

We should note here that while the R1 signal in both white and gray matter is attributable primarily to myelin, it is also influenced by iron concentration^31^. Based on previous findings^32^, we interpreted MDD-related decreases in R1 to reflect reduced myelin rather than reduced iron. Nevertheless, our findings of decreased R1 in MDD should be interpreted with caution until the composition of the R1 signal has been delineated more comprehensively in this disorder. Furthermore, our sample size is not sufficiently powered to assess relations between R1 and psychotropic medication use and anxiety comorbidity; future research should investigate these relations more explicitly and systematically. It will also be important that future research assess long-term clinical features of depression, including the number of prior depressive episodes, more objectively (e.g., by examining medical records) to elucidate more precisely the relations between myelin and depression history and chronicity. The current study did not find evidence of accelerated biological aging of R1 in MDD. Future research should assess relations between age and R1 in samples with wider age ranges. Finally, the functional importance of myelin in MDD needs to be assessed in studies that relate myelin content directly to both brain function and behavior, and to more comprehensive measures of depressive psychopathology.

In conclusion, we found R1 to be reduced in individuals with MDD at the whole-brain level and in the NAcc, compared to healthy individuals. Moreover, individuals with more depressive episodes exhibited greater reductions in LPFC R1 than did individuals with fewer depressive episodes and healthy individuals, even after controlling for levels of whole-brain R1. Our results extend findings from postmortem, animal, and DWI and MTI neuroimaging studies and offer important new evidence of widespread abnormalities in myelin in the brains of depressed individuals. Future research should focus on explicating both the biological mechanisms underlying these abnormalities in R1, including the possible roles of reduced activation and neuroinflammation, and the functional importance of myelin in the pathophysiology of MDD. It will also be important in future studies to examine the utility of R1 as a clinically useful, transdiagnostic, biomarker for the prevention, identification, and treatment of depression and related conditions.

## Methods

### Participants and clinical information

Eighty individuals ages 19–58 years participated in this study: 40 diagnosed with MDD and 40 CTLs. DSM-IV-TR Axis I diagnoses were made by trained interviewers using the Structured Clinical Interview for DSM (SCID^1^). This team of interviewers has demonstrated high inter-rater reliability (i.e., *k*s > 0.9; see ref. 86). Exclusion criteria for both groups of participants were cognitive impairment from head trauma or general medical condition, learning disabilities that interfere with cognitive functioning, alcohol or substance abuse or dependence within the past 6 months, lifetime manic or mixed states, psychosis, significant head trauma (e.g., severe concussion, loss of consciousness for more than 5 min), cardiovascular disease, epilepsy, thyroid disorder, pregnancy, and ferrous material in the body. Potential participants in the CTL group were also excluded if they met criteria for any current or past Axis I disorder or were currently taking psychotropic medication. All participants in the MDD group met diagnostic criteria for a current major depressive episode. The number of previous depressive episodes, current or lifetime anxiety disorder comorbidities, and use of psychotropic medications were recorded for participants in the MDD group. Prior to MRI scanning, participants completed the 10-item Ruminative Response Styles (RRS) questionnaire^87^, a measure of trait depressive rumination. The RRS is composed of two 5-item subscales: brooding (self-critical “moody” pondering) and reflection (emotionally neutral pondering). On the day of MRI data acquisition, participants completed the BDI-II^72^. The BDI-II is a 21-item measure of the severity of depression over the prior two-week period; it assesses levels of various symptom, cognitive, and physical domains related to depression (e.g., sadness, self-criticism, changes in sleep patterns). The BDI-II has been shown to be both reliable and valid^72^. Anhedonia was assessed by summing items 4 and 12 of the BDI-II (“Loss of Pleasure” and “Loss of Interest”, respectively), based on three confirmatory factor analyses involving over 1,000 participants that identified these items as belonging to an “anhedonia” factor^72–74^. The MDD and CTL groups were matched on age, gender, handedness, ethnicity, income, and education. One MDD participant was excluded from the study because of large ventricular volume (46,083.4 mm^3^ and *z*-score = 7.045; *M*/*SE* across all participants without this individual: 6,178.7/381.9 mm^3^); thus, subsequent analyses were conducted using data from 39 MDD and 40 CTL participants. Written informed consent was obtained from each participant, the Stanford University Institutional Review Board approved the study, and all methods were conducted in accordance with relevant guidelines and regulations.

### QMRI data acquisition and estimation of R1

All MRI data were acquired using a 3 T Discovery 750 MRI system (General Electric Healthcare, Milwaukee, WI, USA) with a 32-channel head coil (Nova Medical, Wilmington, MA, USA) housed at the Stanford University Center for Cognitive and Neurobiological Imaging. QMRI data were acquired as in refs 19 and 88. T1 was measured from whole-brain spoiled gradient echo (spoiled-GE) images acquired with four different flip angles (α = 4°, 10°, 20°, and 30°) and repetition time (TR) = 14 ms, echo time (TE) = 2.4 ms, in-plane resolution = 0.938 × 0.938 mm, through-plane resolution = 1 mm, acquisition matrix = 256 × 256, and number of slices = 130.

A series of spin-echo inversion recovery with echo-planar imaging (EPI) read-out (SEIR-EPI) were used for T1 calibration. These scans were collected with a slab-inversion pulse and variable inversion times (50, 400, 1,200, and 2,400 ms). Additional scan parameters were: TE = minimum full, TR = 3 s, in-plane resolution = 1.875 × 1.875 mm, through-plane resolution 4 mm, acquisition matrix = 128 × 128, number of slices median/min/max = 29/26/33 (variable to account for head size). To minimize spatial distortions, EPI read-out was performed using 2 × acceleration.

QMRI data were preprocessed as in refs 19 and 88 using the mrQ software (v.1; available: https://github.com/mezera/mrQ). To estimate T1, it was necessary to correct transmit-coil calibration errors. First, low-resolution T1 was estimated from the SEIR-EPI images using the method described in ref. 89. Next, the low-resolution T1 images were aligned to matched low-resolution spoiled-GE images. Transmit-coil inhomogeneity was then estimated using nonlinear least-squares (NLS) fitting of the MR signal equation^90, 91^ with the low-resolution T1 and multi-flip-angle spoiled-GE images^88^. Then the transmit-coil inhomogeneity and multi-flip-angle spoiled-GE images were used to derive whole-brain T1 maps. This was accomplished using a NLS model fitting approach that minimized differences between the data and signal equation predictions^92^. R1 was then estimated on a voxel-wise basis by computing 1/T1.

### Preprocessing of brain maps

A whole-brain synthetic T1-weighted (sT1w) image was estimated for each participant using the mrQ_T1wSynthesis1 program. Subsequent analyses were conducted using Analysis of Functional NeuroImages (AFNI)^93^. The sT1w images were skullstripped (3dSkullStrip) and intensity normalized (3dUnifize). Next, the individual participant native space sT1w images were transformed and normalized to Montreal Neurological Institute (MNI) space. This was achieved by first using an affine (3dAllineate) transformation and then non-linear (3dQwarp) warp. The linear transformation and non-linear warp were combined and applied to the R1 image (3dNwarpApply). The MNI ICBM 152 version 2009 high-resolution 0.5 × 0.5 × 0.5 mm symmetric template brain^94^ was used as the warp base after resampling it to the native qMR data resolution. Horizontal slice montages of all skull-stripped and normalized brains were visually inspected for quality assurance. In addition, FreeSurfer was used to estimate the volumes of lateral ventricles, the brain segmentation without ventricles, and intracranial volume (eICV) from the sT1w image for all participants^95^. Lateral ventricle volume, brain segmentations, and ICV were compared between groups using independent-sample *t*-tests.

### Regions of interest and extraction of R1

**Figure 1:**
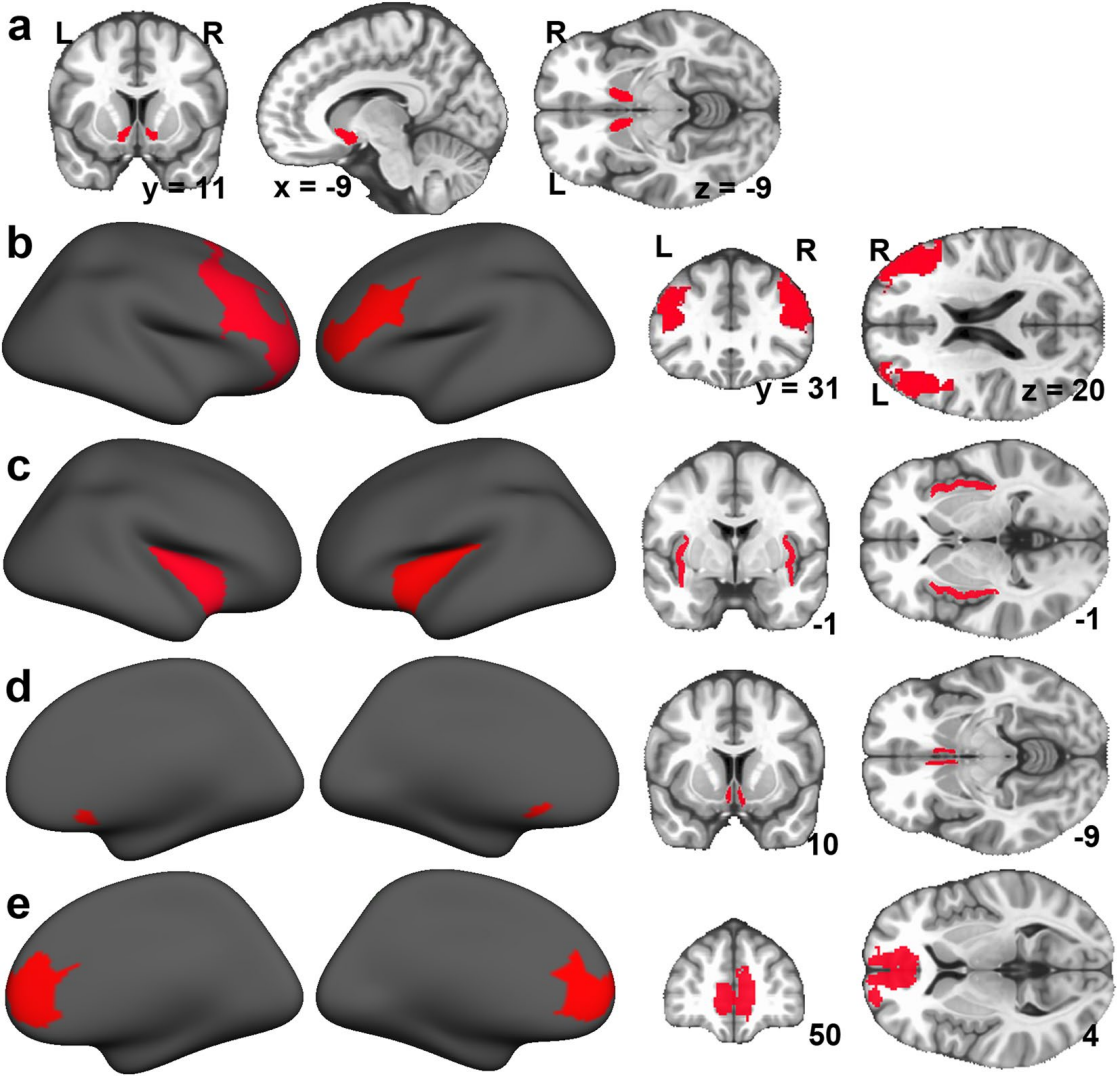
Regions of interest (ROIs). FreeSurfer’s automatic segmentation was used to identify nucleus accumbens (NAcc, **a**)^96^. Lateral prefrontal cortex (LPFC) (**b**) was defined from the Yeo resting-state atlas 7-network atlas^101^ as the prefrontal lateral components of the frontoparietal network. Insula (**c**) was defined using the Desikan^99^ parcellation and subgenual anterior cingulate cortex (sgACC, **d**) was defined using the Destrieux^100^ parcellation, both implemented in FreeSurfer. Medial prefrontal cortex (mPFC, **e**) was defined from the Yeo resting-state atlas 17-network atlas^101^ as the medial prefrontal components of the default mode network. Renderings are depicted on an MNI template. The MNI ICBM 152 version 2009 high-resolution 0.5 × 0.5 × 0.5 mm symmetric template brain^94^ resampled to quantitative magnetic resonance imaging (qMRI) resolution was used as the volumetric image underlay, and volumetric coordinates are in MNI space. Note that both Nacc and insula ROIs were defined in individual native space but are depicted on MNI templates.

We assessed global R1 within a whole-brain MNI mask^94^. Brainstem and cerebellum were removed from the mask because MR acquisitions varied across participants in the coverage of these structures. Whole-brain R1 was computed as the average R1 across voxels within the mask. R1 was assessed in NAcc, LPFC, insula, sgACC, and mPFC using ROI analyses. Both left and right NAcc ROIs were defined for each participant using FreeSurfer’s automatic subcortical segmentation^96^. The FreeSurfer automatic segmentation labels each voxel based on probabilistic information from a manually-labeled training dataset. The procedure is robust to anatomic variability (e.g., ventricular enlargement), has acceptable scan-rescan reliability, and is as accurate as are manual labeling techniques^96–98^. The FreeSurfer automatic segmentation is among the most commonly used segmentation tools and has been applied to the study of many contexts of health and disease. Bilateral insula ROIs were defined for each individual using FreeSurfer’s Desikan anatomical parcellation (aparc + aseg.mgz values 1025 and 2035 for left and right, respectively)^99^. Similarly, the sgACC ROI was identified using FreeSurfer’s Destrieux anatomical parcellation (aparc.a2009s + aseg.mgz values 11132 and 12132 for left and right, respectively)^97, 100^. Given the considerable variability across individual sgACC parcellations in the inclusion of lateral aspects of subcallosal sulcus and gyrus, we defined the sgACC from the FSL MNI template brain rather from individual participant parcellations. In addition, individual sgACC parcellations are affected by the relatively low quality segmentation of gray and white matter in the medial aspects of prefrontal cortex. The FreeView program was used to visually inspect all FreeSurfer segmentations and parcellations for major errors. See Fig. 1 for NAcc, insula, and sgACC ROIs in a MNI template brain.

Bilateral LPFC and mPFC ROIs were defined from the Yeo resting-state atlases (Fig. 1)^101^. The Yeo atlases were defined by clustering the correlations of voxel-wise timecourses from 1,000 resting-state fMRI datasets. LPFC and mPFC ROIs were identified as anatomically isolated regions (“splits”) from larger resting-state networks using a connected component analysis computed on the MNI spherical inflation that incorporated network boundaries, sulcal patterns, and confidence maps (as in ref. 102). Specifically, in each hemisphere, the LPFC ROI was defined as the prominent lateral-frontal split of the frontoparietal network (also referred to as the executive control network; #6) within the 7-network atlas. The LPFC ROI is the central node of the distributed cognitive control network that has been examined using task-based and rest experiments (e.g. refs 103–105). The mPFC ROI was defined from the medial prefrontal split of the default mode network (#16) within the Yeo 17-network atlas (Fig. 1).

**Figure 2:**
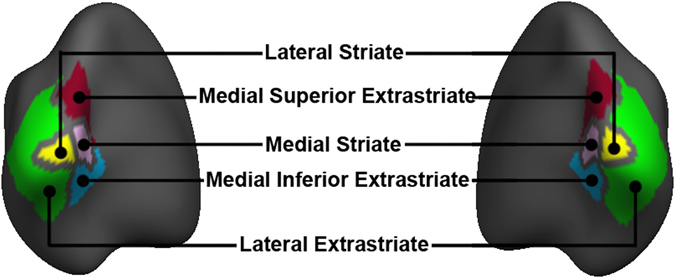
Control regions of interest (ROIs). Five control ROIs (5 per hemisphere) were assessed: lateral striate, lateral extrastraite, medial superior extrastriate, medial striate, and medial inferior extrastriate cortex. These ROIs were defined from the visual network splits of the Yeo resting-state atlas 17-network atlas^101^. Renderings are depicted on an MNI template from a posterior-medial-inferior viewing angle.

Finally, we assessed group R1 in five control regions that are not posited to be involved in MDD. For this purpose we the chose the functionally defined ten visual network splits (five per hemisphere) of the Yeo 17-network atlas^101^. This included lateral striate and extrastriate cortex, and medial striate and inferior and superior extrastriate cortex ROIs (Fig. 2).

### Between-group analyses

We computed average R1 across voxels within each ROI for each individual. We conducted general linear models (GLMs) repeated over hemisphere to assess group (e.g., MDD vs. CTL, more depressive episodes MDD subgroup vs. fewer depressive episodes MDD subgroup) differences in R1 in the NAcc, sgACC, mPFC, insula, LPFC, and the visual and somatosensory network control regions. Given group differences in whole-brain R1, we covaried whole-brain R1 level in these ROI GLMs to assess whether group differences in R1 in specific regions are due to differences in whole-brain levels of R1.

### Relations between R1 and current severity of MDD, anhedonia, and rumination

We examined whether current severity of MDD, as assessed by the BDI-II, was related to R1 at the whole-brain level, and in the NAcc, sgACC, insula, and LPFC. We also examined the relation of NAcc R1 with scores on the anhedonia factor of the BDI-II, and the relation of LPFC, sgACC, and mPFC R1 with levels of rumination, measured by the RRS brooding and reflection subscales. We computed Pearson correlations between R1 and current depression severity and rumination. We used Spearman rank correlation in correlational analysis involving the anhedonia factor because the distribution of scores was non-normal (Lilliefors test: *p* < 0.001).

### Exploratory analyses relating psychotropic medication use and anxiety comorbidity to R1

We conducted exploratory analyses to examine, first, whether MDD participants with a comorbid current anxiety disorder (i.e., generalized anxiety disorder, social anxiety disorder, panic disorder, agoraphobia, obsessive compulsive disorder, or post-traumatic stress-disorder) differ from MDD participants who did not have a comorbid anxiety disorder, and second, whether MDD participants who were taking psychotropic medications (e.g., selective serotonin reuptake inhibitor (SSRI), serotonin–norepinephrine reuptake inhibitor (SNRI), tricyclic antidepressants) differ from MDD participants who were not taking psychotropic medications.

### Exploratory analyses assessing LPFC subregions

**Figure 3:**
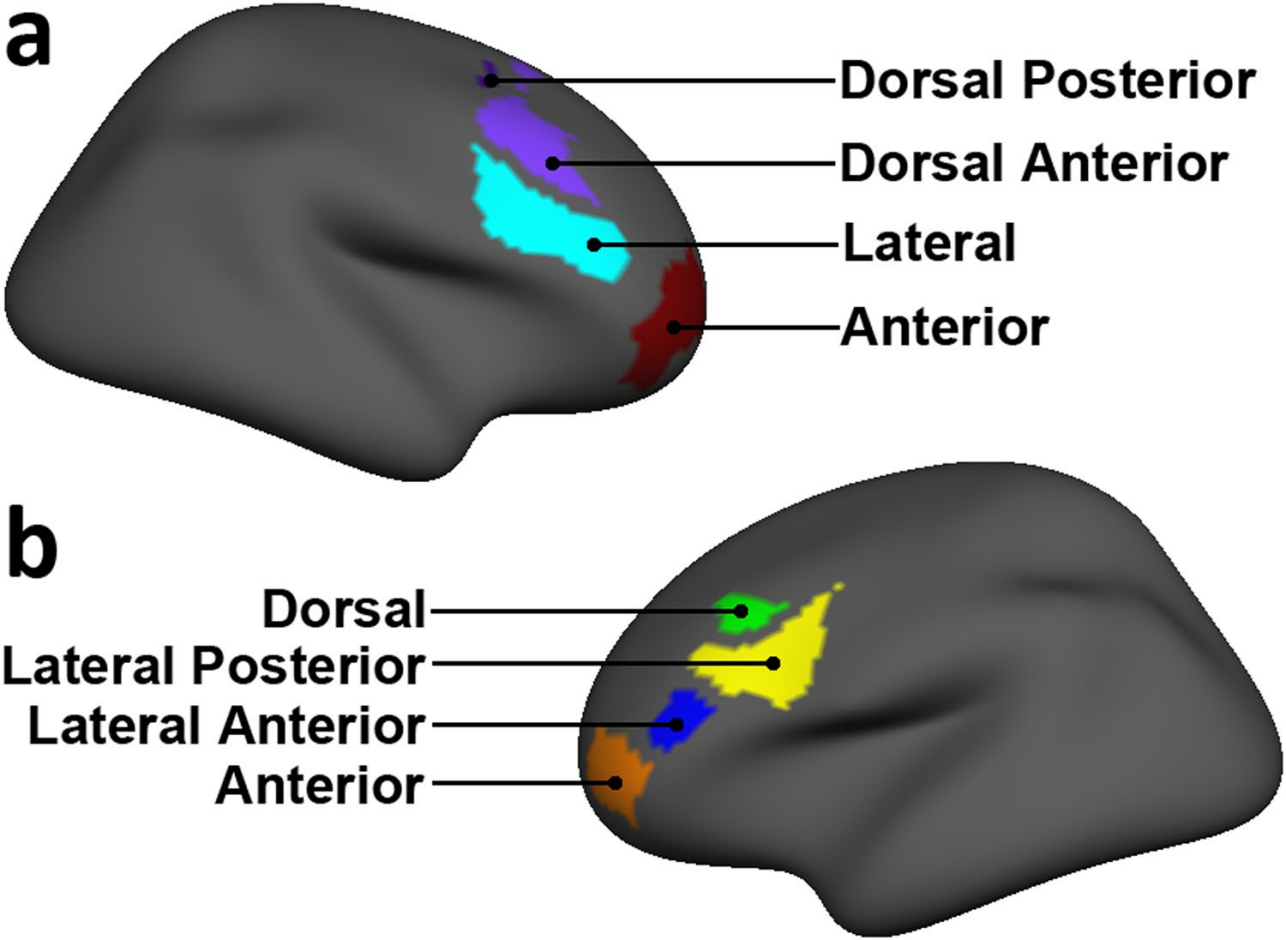
Lateral prefrontal cortex (LPFC) subregions. Renderings of LPFC subregions used in exploratory analyses of R1. The subregions were defined from LPFC splits of the Yeo resting-state atlas 17-network atlas^101^. Subregions are depicted for both right (**a**) and left (**b**) hemispheres. Note bilateral inconsistencies in LPFC subregional architecture. Renderings are depicted on an MNI template.

We conducted exploratory analyses to assess whether subregions of LPFC ROI are differentially myelinated in MDD. LFPC subregions were defined from the Yeo 17-network atlas^101^ in a manner analogous to the LPFC from the Yeo 7-network atlas. Subregions of LPFC in the left hemisphere included anterior, dorsal, lateral anterior, and lateral posterior subregions and, for the right hemisphere: anterior, lateral, dorsal anterior, and dorsal posterior subregions (Fig. 3). We used independent-samples *t*-tests to assess group differences in R1 for the exploratory analyses examining subregions of LPFC. We took this approach to analyzing subregions of LPFC because left and right LPFC subregions are hemisphere-specific with unique spatial coverage as defined by the Yeo 17-network atlas^101^ (Fig. S1); consequently, we could not use an analysis with hemisphere as a repeated measure.

### Relations between R1 and age

Evidence suggests that MDD is characterized by accelerated biological aging^106–110^. As in Sacchet *et al*., we used permutation tests to assess whether the MDD and CTL groups exhibited significantly different relations between age and R1 in NAcc, LPFC, insula, sgACC, and mPFC^106^. First, for a given region, we computed the slopes between age and R1 for each group. Then we computed the difference between slopes. Next, we randomly assigned group labels and recomputed slopes for these permuted data. We repeated this procedure 100,000 times, resulting in a null distribution. Finally, we computed a *p*-value by comparing the original slope difference to this distribution. That is, we defined the *p*-value as the number of permuted datasets that exhibited a larger or smaller slope difference (whichever was smaller) divided by the total number of permutations and multiplied by two for a two-tailed test. We repeated this procedure for each ROI.

## Electronic supplementary material

Supplementary Information
