# Supplementary material for: Myelination of the brain in Major Depressive Disorder: An in vivo quantitative magnetic resonance imaging study
Source: Sci Rep. 2017 May 19;7:2200. doi: 10.1038/s41598-017-02062-y (PMC5438403; doi:10.1038/s41598-017-02062-y)
Supplement: Supplementary file 1 — Supplementary Information [file 41598_2017_2062_MOESM1_ESM.pdf]

Supplementary Information

Myelination of the brain in Major Depressive Disorder: An *in vivo* quantitative magnetic resonance imaging study

Matthew D. Sacchet, Ph.D.[1]*; Ian H. Gotlib, Ph.D.[2,3]

1. Department of Psychiatry and Behavioral Sciences, Stanford, CA 94305
2. Department of Psychology, Stanford University, Stanford, CA 94305
3. Neurosciences Program, Stanford University, Stanford, CA 94305

Supplementary Results

Exploratory analyses relating psychotropic medication use and anxiety comorbidity to R1

We conducted exploratory analyses to assess the effects of psychotropic medication use on whole-brain, NAcc, LPFC, insula, sgACC, and mPFC R1. We formed two subgroups of MDD participants based on current psychotropic medication use (medicated MDD: N=11; unmedicated MDD: N=28). The medicated MDD subgroup did not differ from the unmedicated MDD subgroup with respect to BDI scores ($t(37)=0.494$, $p=0.624$, Cohen's $d=0.18$), or the number of prior depressive episodes ($\chi^2=0.022$, $p=0.883$, $\varphi=0.02$). The unmedicated MDD subgroup had greater whole-brain R1 than did the medicated MDD subgroup ($t(37)=2.29$, $p=0.028$, Cohen's $d=0.83$); NAcc, LPFC, insula, sgACC, and mPFC R1 did not differ as a function of medication subgroup (see Table S1 for complete results). Subsequent t-tests indicated that the medicated MDD subgroup also had lower whole-brain R1 than did participants in the CTL group ($t(49)=3.19$, $p=0.002$, Cohen's $d=1.11$; M/SE: medicated MDD=0.749/0.005; CTL=0.768/0.003); in contrast, the unmedicated MDD subgroup did not differ from the CTL group in whole-brain R1 ($t(66)=1.09$, $p=0.281$, Cohen's $d=0.27$; M/SE: unmedicated MDD=0.754/0.003; CTL=0.768/0.003).

We conducted additional exploratory analyses to assess the effects of anxiety comorbidity on R1. The MDD subgroup with anxiety comorbidity had higher severity of depression than did the MDD subgroup without anxiety comorbidity (BDI: ($t(37)=3.34$, $p=0.002$, Cohen's $d=1.07$; M/SE: MDD with comorbid anxiety=34.68/2.29; MDD without comorbid anxiety disorder=22.82 /2.75); the two subgroups did not differ in the number of depressive episodes ($\chi^2=0.00$; $p=0.987$, $\varphi=0.00$). The subgroup of MDD participants with anxiety comorbidity (N=22) did not differ from the subgroup of MDD participants without anxiety comorbidity (N=17) with respect to levels of whole-brain, NAcc, LPFC, insula, or mPFC R1 (Table S2). The MDD subgroup with anxiety disorder comorbidity had higher sgACC R1 than did the MDD subgroup without anxiety comorbidity ($t(37)=2.07$, $p=0.045$, Cohen's $d=0.67$). Subsequent GLMs (controlling for whole-

brain R1) indicated that the MDD subgroup without anxiety disorder comorbidity had lower sgACC R1 than the CTL group ($F(1)=7.57$, $p=0.008$, partial $\eta^2=0.123$; *EMM*/*SE*: MDD without anxiety disorder comorbidity=0.604/0.006, CTL=0.623/0.004), which did not differ in sgACC R1 from the MDD subgroup with anxiety disorder comorbidity ($F(1)=0.02$, $p=0.893$, partial $\eta^2=0.00$; *EMM*/*SE*: MDD with anxiety disorder comorbidity=0.622/0.005, CTL=0.623/0.003).

Supplementary Tables

Table S1: Exploratory analysis assessing the impact of psychotropic medication use on R1.

	**Unmedicated MDD**		**Medicated MDD**				
**ROI**	***M***	***SE***	***M***	***SE***	***t*-statistic**	***p*-value**	**Cohen's *d***
Whole-brain	0.764	0.003	0.749	0.005	2.29	0.028[a]	0.83
NAcc	0.633	0.005	0.641	0.006	-0.84	0.406	0.32
LPFC	0.738	0.004	0.723	0.007	1.88	0.068	0.66
Insula	0.629	0.003	0.625	0.005	0.87	0.391	0.29
sgACC	0.616	0.006	0.596	0.010	1.83	0.075	0.64
mPFC	0.729	0.005	0.720	0.010	0.89	0.380	0.29

ROI=region of interest; *M*=mean; *SE*=standard error; NAcc=nucleus accumbens; LPFC=lateral prefrontal cortex; sgACC=subgenual anterior cingulate cortex; mPFC=medial prefrontal cortex; [a]=significant at $p<0.05$.

Table S2: Exploratory analysis assessing the impact of anxiety comorbidity on R1.

	**MDD without comorbid anxiety disorder**		**MDD with comorbid anxiety disorder**				
**ROI**	***M***	***SE***	***M***	***SE***	***t*-statistic**	***p*-value**	**Cohen's *d***
Whole-brain	0.757	0.004	0.761	0.004	0.65	0.523	0.21
NAcc	0.641	0.005	0.631	0.006	1.20	0.239	0.39
LPFC	0.731	0.005	0.737	0.005	0.80	0.431	0.26
Insula	0.624	0.003	0.631	0.003	1.41	0.168	0.46
sgACC	0.599	0.008	0.619	0.006	2.07	0.045[a]	0.67
mPFC	0.718	0.006	0.733	0.006	1.71	0.095	0.56

ROI=region of interest; *M*=mean; *SE*=standard error; NAcc=nucleus accumbens; LPFC=lateral prefrontal cortex; sgACC=subgenual anterior cingulate cortex; mPFC=medial prefrontal cortex; [a]=significant at $p<0.05$.

Table S3: R1 assessment of subregions of lateral prefrontal cortex (LPFC). General linear models (GLMs) were used to assess the effect of group in regional R1. Note that the hemispheric uniqueness of LPFC subregions precluded GLMs with hemisphere as a repeated measure.

	MDD		CTL				
**ROI**	***M/EMM***	***SE***	***M/EMM***	***SE***	***F***	***p*-value**	**Partial η^2**
**Without whole-brain R1 covariate**							
L anterior LPFC	0.742	0.005	0.754	0.005	2.55	0.114	0.03
L dorsal LPFC	0.669	0.007	0.689	0.007	4.87	0.030[b]	0.06
L lateral anterior LPFC	0.764	0.008	0.773	0.008	0.76	0.387	0.01
L lateral posterior LPFC	0.841	0.006	0.855	0.006	2.85	0.095	0.04
R anterior LPFC	0.745	0.005	0.747	0.005	0.10	0.750	0.00
R dorsal anterior LPFC	0.630	0.005	0.643	0.005	3.03	0.086	0.04
R dorsal posterior LPFC	0.840	0.013	0.823	0.013	0.85	0.360	0.01
R lateral LPFC	0.757	0.005	0.765	0.005	1.38	0.244	0.02
**With whole-brain R1 covariate[a]**							
L anterior LPFC	0.746	0.005	0.751	0.005	0.56	0.455	0.01
L dorsal LPFC	0.672	0.006	0.686	0.006	2.35	0.129	0.03
L lateral anterior LPFC	0.767	0.008	0.770	0.007	0.07	0.799	0.00
L lateral posterior LPFC	0.845	0.006	0.851	0.005	0.58	0.448	0.01
R anterior LPFC	0.749	0.005	0.744	0.005	0.53	0.471	0.01
R dorsal anterior LPFC	0.634	0.005	0.639	0.005	0.60	0.440	0.01
R dorsal posterior LPFC	0.843	0.013	0.819	0.013	1.67	0.201	0.02
R lateral LPFC	0.760	0.004	0.762	0.004	0.07	0.788	0.00

MDD = Major Depressive Disorder group; CTL = healthy control group; R = right; L = left; ROI = region of interest; *M*/*EMM* = mean or estimated marginal mean, EEM computed when covarying for whole-brain R1; *SE* = standard error; *F* = general linear model *F*-statistic for the effect of group; *t*-statistic = *t*-statistic from two-sample *t*-test between groups; [a]=uncorrected *p*-values; [b]=uncorrected *p*-value<0.05; LPFC = lateral prefrontal cortex.

Table S4: Exploratory analyses of number of prior episodes of Major Depressive Disorder and lateral prefrontal cortex (LPFC) subregional R1. Median split was used to create two groups of MDD participants based on the number of prior depressive episodes. The more episodes group included individuals with 6 or more episodes (*N* = 17), and the fewer group 5 or less episodes (*N* = 22).

	More Episodes		Fewer Episodes				
**ROI**	***M***	***SE***	***M***	***SE***	***t*-statistic**	***p*-value**	**Cohen's *d***
L anterior LPFC	0.737	0.008	0.747	0.005	1.00	0.326	0.31
L dorsal LPFC	0.653	0.007	0.681	0.010	2.31	0.027*	0.77
L lateral anterior LPFC	0.763	0.011	0.765	0.011	0.13	0.898	0.04
L lateral posterior LPFC	0.832	0.009	0.848	0.008	1.23	0.227	0.40
R anterior LPFC	0.735	0.008	0.752	0.007	1.59	0.121	0.51
R dorsal anterior LPFC	0.624	0.009	0.634	0.006	0.95	0.349	0.30
R dorsal posterior LPFC	0.850	0.021	0.832	0.018	0.66	0.515	0.21
R lateral LPFC	0.748	0.007	0.764	0.006	1.87	0.069	0.60

ROI = region of interest; *M* = mean; *SE* = standard error; L = left; R = right; LPFC = lateral prefrontal cortex; * = significant at $p < 0.05$.
